# HIV/HCV Co-infection, Liver Disease Progression, and Age-Related IGF-1 Decline

**DOI:** 10.20411/pai.v2i1.183

**Published:** 2017-03-03

**Authors:** Jeffrey Quinn, Jacquie Astemborski, Shruti H. Mehta, Gregory D. Kirk, David L. Thomas, Ashwin Balagopal

**Affiliations:** 1 Department of Medicine, Johns Hopkins University School of Medicine, Baltimore, Maryland-; 2 Department of Epidemiology, Johns Hopkins Bloomberg School of Public Health, Baltimore, Maryland

**Keywords:** IGF-1, HIV-1, Hepatitis C, Fibrosis, Liver

## Abstract

**Background:**

We have previously reported that persons co-infected with HIV and hepatitis C virus (HCV) had liver disease stages similar to HIV-uninfected individuals who were approximately 10 years older. Insulin-like growth factor 1(IGF-1) levels have long been known to decline with advancing age in humans and non-humans alike. We examined whether HIV infection affects the expected decline in IGF-1 in persons with chronic hepatitis C virus (HCV) infection and if that alteration in IGF-1 decline contributes to the link between HIV, aging, and liver disease progression.

**Methods:**

A total of 553 individuals with HCV infection were studied from the AIDS Linked to the Intravenous Experience (ALIVE) cohort for whom more than 10 years of follow-up was available. Serum IGF-1 levels were determined by ELISA and evaluated according to baseline characteristics and over time by HIV status and liver disease progression. Linear regression with generalized estimating equations was used to determine whether IGF-1 decline over time was independently associated with liver disease progression.

**Results:**

Baseline IGF-1 levels were strongly associated with age (*P* < 0.0001) but not with gender or HIV infection. Levels of IGF-1 declined at a rate of -1.75 ng/mL each year in HCV mono-infected individuals and at a rate of -1.23 ng/mL each year in HIV/HCV co-infected individuals (*P* < 0.05). In a multivariable linear regression model, progression of liver fibrosis was associated with HIV infection and age, as well as with a slower rate of IGF-1 decline (*P* = 0.001); however, the rate of IGF-1 decline did not alter the strength of the associations between HIV, liver disease, and age.

**Conclusions:**

The normal decline in IGF-1 levels with age was attenuated in HIV/HCV co-infected individuals compared to those with HCV mono-infection, and slower IGF-1 decline was independently associated with liver disease progression.

**STANDFIRST**

We measured insulin-like growth factor-1 (IGF-1) levels longitudinally in 553 injection drug users who had liver disease staging with transient elastography, and we found that the decline of IGF-1 over time was strongly associated with liver disease progression. However, our findings did not explain how HIV worsens liver disease.

## INTRODUCTION

People living with HIV (PLWH) infection who have access to combination antiretroviral therapy (cART) are largely protected from AIDS-related mortality [1]. It has been increasingly recognized, however, that PLWH may still suffer from complications of prolonged inflammation that are consistent with premature aging [2–5]. With some conditions it has been difficult to establish if HIV causes these complications or rather if this population is more likely to have other disease risk factors, for example, tobacco use [6]. We have previously reported that, even after adjusting for other risk factors, individuals who were co-infected with HIV and hepatitis C virus (HCV) had liver disease stages equivalent to those who were uninfected with HIV but approximately ten years older [7]. However, it remains unclear how both aging and HIV adversely affect liver disease and whether those mechanisms are related or independent.

Insulin-like growth factor-1 (IGF-1) is chiefly produced in the liver, released in abundance into the blood, and is then delivered to distal tissues to increase protein synthesis [8]. After a rise in production during puberty, serum IGF-1 levels decrease with age [9]. Whereas the implications of decreasing serum levels of IGF-1 in humans are not clear, studies in mice have demonstrated that decreases in IGF-1 levels are associated with increased longevity brought about by moderate caloric depletion [10]. Indeed, in separate murine experiments, longevity among mice that overexpress sirtuins, genes that are known to regulate lifespan, was associated with decreases in IGF-1 levels [11]. An independent study exploring determinants of aging in humans found loss of function mutations in the IGF-1 pathway, and the resulting low IGF-1 levels, to be associated with longevity in in humans [12, 13]. These findings in both humans and non-human model systems suggest that while aging itself is associated with lower IGF-1, a failure to reduce IGF-1 with age may have unfavorable health consequences.

We tested the hypothesis that HIV promotes liver disease progression in people with HCV co-infection by altering the decline in IGF-1 levels. We examined serum IGF-1 levels over time in a cohort of HCV-infected individuals for whom liver disease progression was carefully measured and in whom we previously reported strong associations between HIV, aging, and liver disease.

## METHODS

Samples were obtained from 553 persons enrolled in the AIDS Linked to the Intravenous Experience (ALIVE) cohort for whom HCV, HIV, and the progression of liver disease were well characterized, and more than 10 years of follow-up was available. The ALIVE cohort was formed in 1988-89 and includes injection drug users aged 18 years or older recruited through street-based efforts. The ALIVE cohort has been continually approved by the Johns Hopkins Institutional Review Board (Bloomberg School of Public Health), and all participants gave written informed consent. Cohort members are evaluated at semi-annual visits using questionnaires, examinations, and blood tests. Beginning in 2006, liver fibrosis was evaluated using elastography as described [7]. Compared to all 2,941 participants initially enrolled in the cohort, these 553 individuals were younger (50.6 versus 51.8 years), less likely to be HIV positive (31.4% vs 37.7%), more likely to be HCV RNA positive (86.3% versus 79.5%), and more likely to be female (27.8% vs 24.7%) (*P* > 0.05 for all comparisons). Participants were selected based on earliest enrollment in the cohort to maximize the amount of time in which IGF-1 levels could be measured. Their HIV status was ascertained by ELISA testing followed by Western blot to confirm positivity, and all participants had follow-up plasma HIV RNA levels performed by quantitative PCR (qPCR) using the COBAS AmpliPrep/COBAS Taqman HIV-1 Monitor test, version 2 (Roche Diagnostics, Indianapolis, Indiana). Similarly, HCV infection was documented by anti-HCV antibody testing with the Ortho HCV ELISA 3.0/2.0 assay (Ortho Clinical Diagnostics, Raritan, NJ) followed by HCV RNA levels determined by qPCR using the Abbott RealTime HCV assay (Abbott Molecular, Des Plaines, Illinois) for determination of chronically infected individuals.

The progression of liver disease was ascertained by elastography. From 2006-2014, liver disease was staged as liver stiffness, as measured by transient elastography (TE) using a FibroScan machine that measures the velocity of a shear wave propagating through the liver, as previously reported [14]. Certified operators were trained by the manufacturer and performed 8 discrete validated measurements that were expressed in kilopascals (kPa). The TE success rates were greater than 60% (number of validated measurements divided by the total number of measurements) and had limited variability (interquartile range [IQR] of measures divided by the median value < 0.30). The median value from each valid examination was used for analysis. Results for TE were obtained at six-month intervals from 2006-2014 [14].

Baseline samples (t_1_) for each participant were selected based on earliest enrollment in the cohort to maximize the amount of time in which IGF-1 levels could be measured. A second sample (t_2_) was obtained from each participant that was selected to coincide with the earliest TE measurement. The most recently available sample was selected as the third sample (t_3_). Sera were assayed by ELISA for IGF-1 (R&D Systems, Inc., Minneapolis, MN) at each time point according to the manufacturer's protocol.

Participants were stratified into groups by viral infection status: HCV mono-infected participants, HIV/HCV co-infected participants, and those without HIV or HCV. Summary statistics included age, gender, HCV status by RNA positivity and antibody status, plasma HCV RNA level, HIV status by RNA, plasma HIV RNA level, and CD4^+^ T cell count. Initially, baseline correlates of IGF-1 levels were examined by univariable linear regression models. The results of IGF-1 were also transformed by calculating the natural logarithm, but none of the associations were changed (data not shown) and the overall model fit was not substantially improved. To account for the interval of time from baseline to the initial liver stiffness evaluation, a rate of change in IGF-1 levels was calculated for each individual using the formula: [IGF-1(t_2_) – IGF-1(t_1_)]/[t_2_-t_1_]. Rates of change for the full interval were calculated separately by substituting t_3_ for t_2_ in the above formula. These values were not significantly different and are not shown since the exposure (t_3_) was later than the liver stiffness evaluation (t_2_). Differences in the rate of IGF-1 decline by HIV status were compared by Wilcoxon rank sum test.

To assess the decline in IGF-1 levels as a determinant of liver stiffness, models of liver stiffness were constructed with the rate of IGF-1 decline considered as an explanatory variable. Liver stiffness was analyzed using gamma models of untransformed values truncated at 20 kPa, using the log-link function as previously described [7]. The intra-person correlation of repeated measurements of IGF-1 was accounted for by using generalized estimating equations assuming an exchangeable correlation structure, based on detailed exploration of the data.

## RESULTS

Among the 553 individuals who were studied, the median (IQR) age at baseline was 38.3 years (34.4-42.6), 158 (28.6%) were female, 536 (96.9%) were black, 63 were HIV and HCV uninfected (11.4%), 361 (65.3%) had HCV mono-infection, and 129 (23.3%) had HIV co-infection (Table 1). At baseline (t_1_), the median (IQR) IGF-1 level was 107.5 (80.4-134.5) ng/mL. Age was strongly associated with lower IGF-1 level (*P* < 0.0001). No associations were detected between baseline IGF-1 level and gender, race, HIV status, or HCV status.

**Table 1.** Characteristics of the 553 study participants and baseline correlates of IGF-1^a^

Correlate	N (%)	Estimate	95% Confidence interval	*P*-value
**Age (years)^b^**				
<34	131 (23.7)	1.00	-	-
34-47	134 (24.2)	−11.44	−21.22, −1.67	0.02
38-42	161 (29.1)	−19.00	−28.36, 9.64	<0.0001
≥ 43	127 (23.0)	−20.89	−30.80, −10.93	<0.0001
**Sex**				
Male	395 (71.4)	1.00	−	−
Female	158 (28.6)	0.52	−7.11, +8.14	0.89
**Race**				
Non-black	17 (3.1)	1.00	−	−
Black	536 (96.9)	−2.61	−22.56, +17.34	0.80
**HCV antibody**				
Negative^c^	63 (11.4)	1.00	−	−
Positive	490 (88.6)	−8.33	−19.15, +2.49	0.13
**HCV RNA**				
Negative^c^	63 (11.4)	1.00	−	−
HCV positive/RNA non-detected	66 (12.1)	−2.50	−16.79, +11.80	0.73
HCV positive/RNA detected	418 (76.4)	−9.19	−20.16, +1.78	0.10
**HIV antibody**				
Negative	424 (76.7)	1.00	−	−
Positive	129 (23.3)	−1.04	−9.19, +7.10	0.80
**HIV RNA**				
Undetectable	424 (76..7)	1.00	−	−
<4 log_10_ cp/mL	23 (4.2)	0.36	−16.61, +17.33	0.97
≥ 4 log_10_ cp/mL	20 (3.6)	−11.37	+29.51, +6.76	0.22
Missing	86 (15.5)	−	−	−
**CD4^+^ T cell count (cell/uL)**				
HIV uninfected	424 (76.7)	1.00	−	−
≥ 500	46 (8.3)	4.67	−7.68, +17.02	0.46
200-499	54 (9.8)	−11.76	−23.26, −0.26	0.05
<200	25 (4.5)	1.33	+15.05, +17.70	0.87
Missing	4 (0.007)	−	−	−

Abbreviations: cp/mL, copies per milliliter

^a^ Indicated are estimates of associations of baseline IGF-1 levels with covariates also determined at baseline unless otherwise noted.

^b^ HCV RNA measurements are represented at t_2_

^c^ All 63 individuals negative for HCV antibody and RNA are also negative for HIV antibody and RNA

Levels of IGF-1 were assessed over a span of a median (range) of 16 (15-17) years per person overall: 16.22 (9.88 – 16.96) years for HIV positive and 16.21 (4.44 – 16.96) years for HIV negative (*P* = 0.63). Levels of IGF-1 declined with age (*P* < 0.0001) and the median (IQR) IGF-1 decline over time was -1.66 ng/mL (-3.23 – -0.26) per year. Notably, the per person IGF-1 decline in the HIV/HCV co-infected population (-1.23 ng/mL) was significantly less than in the HCV mono-infected population (-1.75 ng/mL) (*P* < 0.05).

In this cohort subset, liver stiffness was associated with age, HIV infection status, obesity, and HCV RNA level, as previously described with the larger cohort (Table 2) [7]. In the same model, IGF-1 decline over time was also significantly associated with liver stiffness, independently of HIV status and age (*P* = 0.001, Table 2). However, adding the decline of IGF-1 levels to the model did not significantly change the associations of age, HIV, and liver disease.

**Table 2.** Factors associated with liver stiffness

Model 1			
	Coefficient	95% Confidence interval	*P*-value
**Age, by decade**	0.11	0.42 – 0.18	0.001
**BMI**			
Normal	1.00	-	-
Overweight	0.12	0.07 – 0.17	<0.001
Obese	0.16	0.11 – 0.23	<0.001
**HCV RNA**			
Undetectable	1.00	-	-
<6 log10 IU/mL	0.17	0.04 – 0.31	0.010
≥ 6 log10 IU/mL	0.28	0.17 – 0.40	<0.001
**HBsAg**	0.08	−0.31 – 0.47	0.681
**HIV antibody**	0.13	0.03 – 0.22	0.009

Model 2			
	Coefficient	95% Confidence interval	*P*-value
**Age, by decade**	0.12	0.05 – 0.18	<0.001
**BMI**			
Normal	1.00	-	-
Overweight	0.12	0.07 – 0.16	<0.001
Obese	0.16	0.10 – 0.22	<0.001
**HCV RNA**			
Undetectable	1.00	-	-
<6 log_10_ IU/mL	0.16	0.03 – 0.29	0.014
≥ 6 log_10_ IU/mL	0.26	0.15 – 0.38	<0.001
**HBsAg**	0.11	−0.24 – 0.47	0.526
**HIV antibody**	0.14	0.04 – 0.23	0.005
**Rate of IGF-1 decline^a^**	−0.02	−0.003 – −0.01	0.001

Abbreviations: IU/mL, International units per milliliter HBsAg, Hepatitis B surface antigen;

^a^ A coefficient <0 indicates that individuals with less age-related IGF-1 decline were more likely to have higher liver stiffness values.

## DISCUSSION

In the present study, serum IGF-1 levels were strongly associated with age and declined with age, as expected. We observed a small but significant attenuation in IGF-1 decline in HIV/HCV co-infection compared to mono-infection. In addition, IGF-1 decline was independently associated with liver fibrosis progression, but this decline did not appear to explain how HIV infection “ages” the liver.

IGF-1 has long been appreciated as a marker for biological aging [13]. The age-related decline in IGF-1 levels has been thought to be associated with a decrease in the metabolic needs of aging cells. In a proof-of-concept study, Breese *et al* reported that modest caloric restriction of rats increased longevity and was associated with further decreases in IGF-1 levels compared to normally fed rats [10], thereby linking longevity with IGF-1 decline. The authors further proposed that decreases in IGF-1 levels over time may lead to fewer long-term pathologies [9]. It has also been reported that IGF-1 signaling in cells may directly contribute to aging as evidenced by associations between loss of function mutations in the IGF-1 pathway and longevity in non-human models and in humans [11–13].

Prior studies of IGF-1 in HIV-infected individuals have reported decreased levels in the setting of HIV. However, these were largely performed in the pre-cART era and were focused on AIDS wasting, illustrating the opposite end of the spectrum of IGF-1 related pathologies [8, 15]. In a separate investigation, IGF-1 levels were used to predict incident liver damage in HIV/HCV co-infected patients, illustrating that elevations of IGF-1 were indeed associated with a higher likelihood of having elevated transaminase levels [15], although the authors did not stage liver fibrosis in that study.

The extreme situations of liver cirrhosis and end-stage liver disease have been associated with low IGF-1 levels in both adults and children in cross-sectional studies [16–20]. This finding is likely because of the reduced number of hepatocytes, which produce IGF-1, in the cirrhotic liver with advanced dysfunction [21]. Likewise, HCV RNA and albumin levels follow the same pattern of decline in the terminal stages of liver disease [7]. Notably, in the present study IGF-1 decline was independently associated with HIV status and liver disease.

In the analysis, we failed to detect an association of hepatitis B surface antigen (HBsAg) with liver stiffness after adjusting for other factors. It is important to recognize that the majority of all study individuals (490/553 = 88.6%) were anti-HCV positive. Therefore, since most of those with chronic hepatitis B also had chronic HCV infection, the HBsAg coefficient referred to the added effect of HBV.

There were several challenges in the present study. A possible consequence of our sampling strategy is that we may not have captured participants who succumbed to liver-related deaths within 5 years of enrollment. However, since the time of enrollment was arbitrary within the natural history of a given participant's HIV and HCV infections, we were not systematically biased against studying those with shorter longevity. Moreover, because using IGF-1 values cannot discriminate age differences within 5 years, we would not have been able to determine whether premature aging contributed to an individual's liver-related death if they died within a short time after enrollment. Hence, survivor bias is likely to have a limited effect on our main findings. A further challenge in the present study was in deducing whether changes in IGF-1 decline were the cause or effect of HIV infection and liver disease. In addition, we had only 3 IGF measurements per person to approximate the trajectories of each person, and information could have been missing on visit-to-visit changes between those determinations.

In a well-characterized cohort of individuals who were studied for more than a decade, we found that HIV slowed the decline of IGF-1. The IGF-1 decline, in turn, was associated with liver disease progression, although this association did not explain how HIV worsens liver disease. Future studies should focus on approaches to understanding and restoring this balance.
